# Trends in adolescent psychosomatic complaints: a quantile regression analysis of Swedish HBSC data 1985–2017

**DOI:** 10.1177/14034948221094497

**Published:** 2022-05-07

**Authors:** Björn Högberg, Mattias Strandh, Klara Johansson, Solveig Petersen

**Affiliations:** 1 Department of Social Work, Umeå University, Umeå, Sweden; 2 Centre for Demographic and Ageing Research (CEDAR), Umeå University, Umeå, Sweden; 3 Department of Epidemiology and Global Health, Umeå University, Umeå, Sweden

**Keywords:** Adolescents, children, health complaints, psychosomatic, mental health, temporal trends, HBSC, Sweden

## Abstract

**Background and aims:**

According to recent criticism, survey-based measures of adolescent psychosomatic complaints have poor content validity insofar as they conflate trivial with severe complaints. It is argued that this means that estimates of prevalence and trends in complaints may reflect trivial complaints that are not indicators of health problems. In this study, two observable implications of this criticism were investigated: (a) that self-reported psychosomatic complaints should have a bimodal distribution; and (b) that the increase in complaints over time should be of approximately equal size throughout the distribution of complaints.

**Methods:**

Three decades (1985/1986–2017/2018) of repeated cross-sectional data from the Swedish Health Behaviour in School-aged Children survey were used. Psychosomatic complaints were measured using the screening instrument Health Behaviour in School-aged Children symptom checklist. Histograms, bar charts and quantile regression models were used for the analysis.

**Results and conclusions:**

**With regard to the first implication, the results showed that the distribution of complaints was not bimodal and that there were no clusters of respondents. This suggests that binary categorisations of students can be reductive and conceal important variations across students. With regard to the second implication, the results showed that the increase in complaints was greatest among students who report frequent and co-occurring complaints. This suggests that reports of increasing complaints in adolescents cannot be explained as being primarily due to a greater inclination to report trivial complaints. It is concluded that any conflation of trivial and more severe complaints in surveys of psychosomatic complaints is not reflected in population-based estimates.**

## Background

Several empirical studies have indicated an increase in self-reported psychosomatic and other mental health-related complaints in adolescents in high-income countries, including Sweden [1–7]. The increased prevalence of such complaints has received substantial public and scientific attention, leading to calls for public health interventions [8].

Psychosomatic complaints, also known as psychosomatic symptoms or psychosomatic problems, commonly refer to a combination of subjective psychological health complaints such as sadness and irritability, as well as physical complaints such as headaches and stomach aches [2–4, 9, 10]. Psychosomatic complaints are associated with poor mental health, as captured by indicators of mental disorders [11–13]. In interviews, adolescents also report that these types of complaints are perceived as indicative of their mental health [14]. Hence, trends in psychosomatic complaints are also sometimes referred to as trends in mental ill-health problems [6, 7].

Previous research on trends in psychosomatic complaints has often relied on questionnaire surveys in which respondents are asked how often they have experienced various complaints. One example is the commonly used World Health Organization (WHO) survey Health Behaviour in School-aged Children (HBSC), which measures the presence of psychosomatic complaints in adolescents using a symptom checklist (SCL).

Despite the widespread use of survey-based methods, their content validity was recently questioned by Wickström and Lindholm [15]. Based on an interview study of what the HBSC-SCL items ‘represent’ for 15-year-old adolescents, the authors conclude that these types of items conflate two different sets of complaints: trivial (or ‘everyday’) complaints experienced by the majority, and severe (or ‘deep-seated’) complaints experienced by the minority. They further argue that this conflation means that survey-based estimates may create an unwarranted representation of adolescents as ‘a homogeneous group increasingly suffering from poor mental health’ [15]. Accordingly, the researchers question the validity of population-level estimates of psychosomatic complaints and argue for caution when talking about a high and increasing prevalence of such problems in adolescents [16, 17]. Their criticism has received substantial public attention, with interviews on national TV, radio and in newspapers (e.g. Ungas psykiska hälsa klumpas ihop med vardagliga problem: ‘vi missar målet’) [17]. The criticism may also have far-reaching consequences for the interpretation of the survey-based literature on adolescent psychosomatic complaints. These kinds of measures have inspired extensive public health interventions [8] and it is imperative that such interventions are based on accurate descriptions of the problems they aim to solve.

The study of Wickström and Lindholm [15] was based on interviews with a sample of 41 adolescents, mostly girls, attending two particular schools. Thus, there are reasons to investigate further the relevance of their criticism using population-level data. One way to achieve this is to test whether the observable implications of the argument underlying the criticism are consistent with the response patterns in the population-level data. ‘Observable implications’ here refers to empirical patterns that should be present in the data if this argument is correct [18]. In particular, if the argument underlying the criticism is correct, the following patterns should be expected in surveys on psychosomatic complaints:

- If the distinction between students with trivial (the majority) and severe (the minority) complaints has a substantial impact on the response patterns in surveys, the frequency and co-occurrence of complaints should have a bimodal distribution: there should be a large cluster reporting relatively infrequent complaints and a smaller cluster reporting relatively frequent and co-occurring complaints, with few intermediate cases. Note that frequent and infrequent are to be understood in a relative sense, but there are no predefined cut-off values that separate the two clusters. This first implication relates to the cross-sectional prevalence of complaints.
- If the increase in complaints over time mainly reflects an increase in the prevalence of trivial complaints, the increase should be equally large across the distribution of complaints. An increase in the prevalence of severe complaints would instead imply a disproportionate increase in the proportion of students who report relatively frequent and co-occurring complaints. This second implication relates to the trend in complaints.

Note that these implications only follow from the argument of Wickström and Lindholm [15] if two assumptions are met. The first implication assumes that the frequency and co-occurrence of complaints is positively associated with their severity and negatively associated with the respondent ‘feeling well’, which is the main dividing line between trivial and severe complaints, according to Wickström and Lindholm [15]. This assumption is supported by studies showing that the frequency and co-occurrence of complaints predict clinical depression and suicidal ideation [19, 20], emotional or behavioural functioning [11], as well as impaired function [21, 22]. The second implication assumes that this association is stable over time. This issue has been less thoroughly investigated, although one study found that trends in complaints are congruent with trends in functional impairment in Swedish adolescents [3].

### Aim

Based on the observable implications described above, the aim of this study was to investigate the characteristics of: (a) the cross-sectional distribution; and (b) the temporal trends of psychosomatic complaints (as defined in the HBSC-SCL) in Swedish adolescents aged 15 years.

## Methods

### Data

Swedish data from the HBSC survey were used. HBSC is an international cross-sectional and school-based survey carried out every 4 years in collaboration with the WHO. The aim is to understand the health, health behaviours and life circumstances of children and adolescents aged 11–15 years. The Public Health Agency of Sweden was responsible for the content of the Swedish version of the survey, while Statistics Sweden was responsible for the data collection. All surveys with complete data on the HBSC-SCL were included, namely, those surveys from 1985/1986, 1993/1994, 1997/1998, 2001/2002, 2005/2006, 2009/2010, 2013/2014 and 2017/2018. The 1989/1990 survey used different response options and was excluded from the analysis.

Data were collected through a two-stage cluster design. First, 200–500 schools were randomly sampled with equal probability from all Swedish schools that enrolled students in the 5th, 7th and 9th school years. Second, one school class from each of the school years was randomly sampled from each school and all students in these classes were invited to participate. The invited students and their guardians were given written information about the survey, stating that their participation is confidential and voluntary. Students who verbally consented to participate completed the survey in the classroom.

Response rates at the school level declined from 100% in 1985/1986 to approximately 80% in the 2001/2002 survey through to the 2013/2014 survey, and to 47% in the 2017/2018 survey. The decline in 2017/2018 was due to new regulations that prohibited Statistics Sweden from sending reminders to non-participating schools. A non-response analysis showed no major differences between the sociodemographic characteristics of the respondents in the 2017/2018 survey and those of previous surveys [23]. Response rates at the student level have been stable at approximately 85–90% in all surveys since 1985/1986. The sample was restricted to students in the 9th school year (age 15 years), in keeping with the study by Wickström and Lindholm [15].

### Variables

Psychosomatic complaints were measured using the HBSC-SCL, an instrument developed to capture adolescents’ perceptions of their health, rather than for diagnostic purposes [24]. The respondents were asked how often they have one or more of the following complaints: headaches, stomach aches, backache, feeling low or depressed, being irritable or bad tempered, being nervous, sleeping difficulties, or dizziness. The response options were (4) ‘About every day’, (3) ‘More than once a week’, (2) ‘About every week’, (1) ‘About every month’ and (0) ‘Rarely or never’. Based on these items, an additive scale with a range of 0–32 was constructed, with higher values representing more frequent and co-occurring complaints. The total sum score is called the HBSC-SCL score. The HBSC-SCL has shown adequate reliability and validity [24] and is predictive of emotional problems and suicidal ideation in adolescents [20, 25]. As the focus is on the distribution of the HBSC-SCL scores, no covariates other than survey year were included.

### Analytical strategy

In order to investigate the prevalence of complaints cross-sectionally (implication no. 1), the distribution of HBSC-SCL scores were visualised using histograms. The distribution of the HBSC-SCL score was analysed for all years together. To enable comparisons over time, the years 1985/1986 and 2017/2018 were also analysed separately. In order to investigate temporal trends (implication no. 2), quantile regression models were used. The HBSC-SCL score was the dependent variable and the survey year was the independent variable. Quantile regression models estimate the effect of an independent variable at different percentiles of the dependent variable. Using survey year as the sole independent variable, this corresponded to the position of a student in a specific percentile of the distribution in a specific year. Changes in response patterns for each individual item between 1985/1986 and 2017/2018 were also analysed. This was conducted by determining the proportion of respondents who chose a given response option for a given item in 2017/2018 and dividing it by the corresponding proportion for 1985/1986. The resulting ratio indicates the change in the proportion of respondents who chose that response option for that item.

Two sensitivity analyses were conducted. First, the response options ‘About every day’ and ‘More than once a week’ were collapsed into one category, and ‘About once a week’ and ‘About once a month’ were collapsed into another category, in line with recent recommendations [4]. Second, boys and girls were analysed separately.

## Results

Table I shows that the mean HBSC-SCL score and its standard deviation increased over time for both boys and girls. This means that students on average reported more frequent and co-occurring complaints in the more recent surveys, and also that there was a greater interindividual dispersion in the complaints. The total sample size for all surveys combined was 12,186 students.

**Table I.** Sample characteristics.

Survey year	Sample size 9th school year			HBSC-SCL: mean			HBSC-SCL: standard deviation		
	Girls	Boys	Total	Girls	Boys	Total	Girls	Boys	Total
1985/1986	527	545	1072	8.51	6.58	7.53	5.25	4.26	4.87
1993/1994	557	576	1133	10.62	7.89	9.23	5.95	5.10	5.70
1997/1998	539	601	1140	11.37	8.83	10.03	6.29	6.07	6.30
2001/2002	609	600	1209	12.03	8.20	10.13	6.45	5.49	6.29
2005/2006	765	746	1511	12.70	8.59	10.67	6.71	5.91	6.65
2009/2010	988	1020	2008	12.20	8.03	10.03	6.53	6.07	6.65
2013/2014	1332	1283	2615	13.53	8.78	11.20	6.77	6.36	6.99
2017/2018	782	716	1498	14.19	9.65	12.02	6.63	6.17	6.80
Total/average	6099	6087	12,186	12.25	8.40	10.32	6.62	5.89	6.56

HBSC-SCL: Health Behaviour in School-aged Children symptom checklist. Data from the Swedish version of the Health Behaviour in School-aged Children survey.

**Figure 1.**
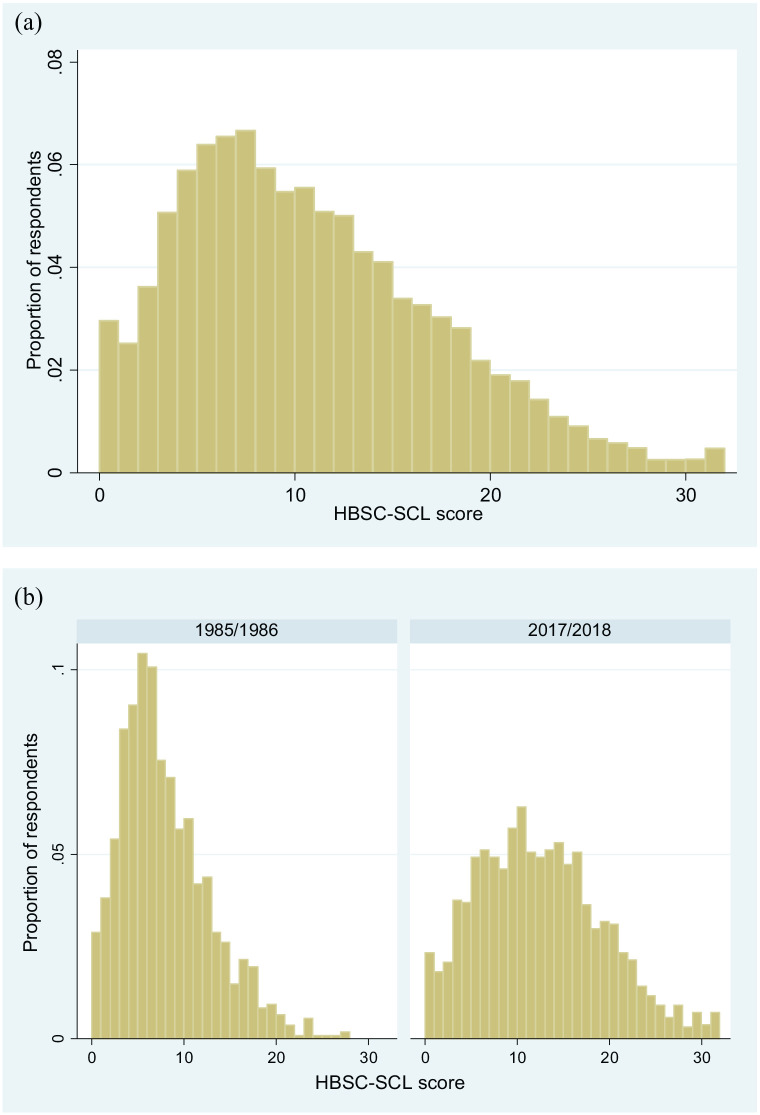
Histogram of (a) the distribution of HBSC-SCL scores in all years combined and (b) in 1985/1986 and 2017/2018 separately. Data from the Swedish version of the Health Behaviour in School-aged Children (HBSC) survey. HBSC-SCL: Health Behaviour in School-aged Children symptom checklist. All years refer to surveys from 1985/1986, 1993/1994, 1997/1998, 2001/2002, 2005/2006, 2009/2010, 2013/2014 and 2017/2018.

Figure 1 addresses observable implication no. 1 by showing the distribution of HBSC-SCL scores. The cross-sectional distributions were not bimodal and did not exhibit any evidence of discontinuities or clusters. Instead, the distributions were smooth and continuous for all samples. Moreover, the distribution for 1985/1986 was strongly positively skewed, while the distribution for 2017/2018 was more fat tailed, meaning that the proportion of students with relatively frequent and co-occurring complaints increased between these surveys.

**Figure 2.**
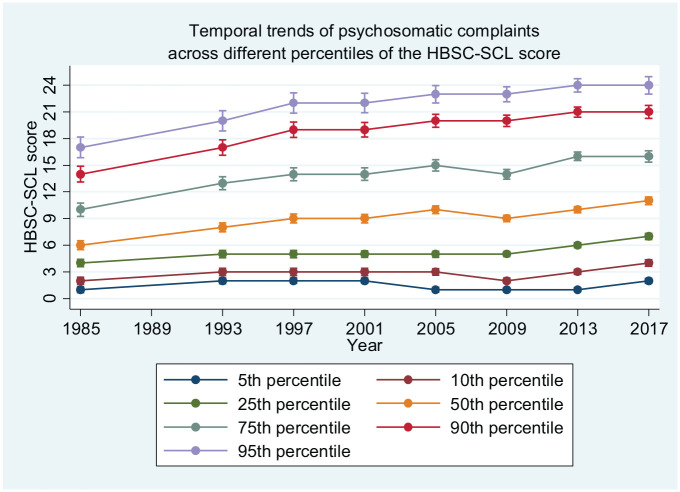
Temporal trends of psychosomatic complaints across different percentiles of the HBSC-SCL score. Data from the Swedish version of the Health Behaviour in School-aged Children (HBSC) survey. HBSC-SCL: Health Behaviour in School-aged Children symptom checklist.

Figure 2 shows percentiles of the HBSC-SCL scores across all years, thereby addressing observable implication no. 2. Each line in the figure depicts the score for an individual student at that percentile. The increase in complaints was stronger at the top of the distribution than at the bottom. The 90th and 95th percentiles increased by seven scale points between 1985/1986 and 2017/2018, while the 5th and 10th percentiles increased by one and two scale points, respectively.

**Figure 3.**
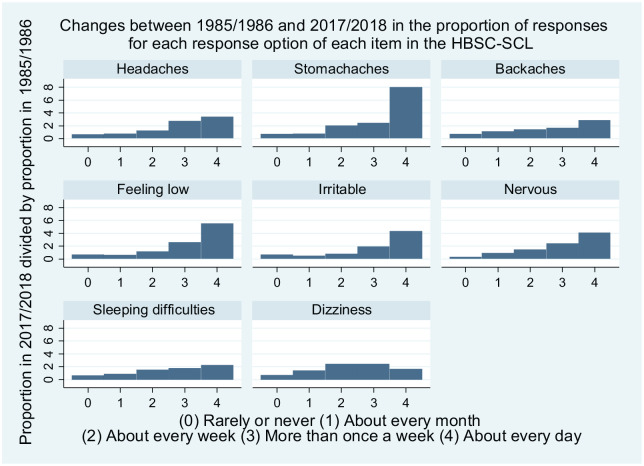
Changes between 1985/1986 and 2017/2018 in the Swedish Health Behaviour in School-aged Children (HBSC) survey, for each item in the HBSC symptom checklist (SCL). The bars show ratios of the proportion of responses for each response option, with the proportion in 1985/1986 as the denominator and the proportion in 2017/2018 as the numerator. Thus the proportion who responded that they had stomach aches ‘About every day’, for instance, increased eight-fold over the period, while the proportion who responded that they ‘Rarely or never’ had stomach aches declined by approximately 25%.

Figure 3 shows how the proportion of students choosing each response option for each item changed between 1985/1986 and 2017/2018. The proportion of students reporting that they experienced a specific complaint ‘About every day’ increased between two (dizziness) and eight (stomach aches) times over the period. Thus the disproportionate increase at the top of the distribution of the HBSC-SCL scores was driven by an increase in the proportion of students reporting complaints ‘About every day’.

### Supplemental analyses

Results from the two sensitivity analyses are presented in the online supplemental materials. First, Supplemental Figure 1(a) and (b) and Supplemental Figure 2 in the online supplemental materials show that the prevalence of complaints and their trends observed after collapsing the response options were qualitatively similar to those presented in the main analysis. Second, Supplemental Figure 3(a) and (b) in the online supplemental materials show that the results were similar for both genders: the distribution of complaints was smooth and the increase in complaints was stronger at the top of the distribution, although the gap between lower and higher percentiles was larger in boys.

## Discussion

The aim of this study was to investigate the characteristics of the cross-sectional distribution and the temporal trends of psychosomatic complaints (as defined in the HBSC-SCL) in Swedish adolescents aged 15 years. The results showed that the distribution of complaints was smooth, and that there was a disproportionate increase in the proportion of students who report relatively frequent and co-occurring complaints.

These results suggest that the previously published criticism of the content validity of the HBSC-SCL, as presented by Wickström and Lindholm [15], is inconsistent with the population-level data. The criticism presented in their study had two key observable implications. The first implication was that the frequency and co-occurrence of complaints should have a bimodal distribution. In contrast to this, the results of the current study showed no indications that there are distinct groups of respondents in terms of their response patterns to the items included in the HBSC-SCL. This, in turn, suggests that binary categorisations of students, such as distinctions between students with trivial versus severe complaints [17], are reductive and conceal important variations across students. Moreover, if there are no distinct groups of students in this regard, there is no reason to assume that measuring psychosomatic health based on the frequency and co-occurrence of complaints, as is the case with the HBSC-SCL, systematically misclassifies students based on such group affiliations. Thus it is concluded that the afore-mentioned criticism should not discourage researchers from using the HBSC-SCL in the analyses of prevalence or trends in psychosomatic complaints.

The second implication was that the increase in complaints over time should be approximately equally spread across the distribution of complaints. In contrast to this, the results showed that the increase in complaints between 1985/1986 and 2017/2018 was substantially more pronounced at the top than at the bottom of the distribution. The overall increase was driven by a disproportionate increase in the proportion of students who report relatively frequent complaints. The increase was particularly high in those students who experienced complaints ‘About every day’, which would approximately correspond to a disproportionate increase in the proportion of students with severe complaints. Thus the distribution of complaints has become more polarised insofar as there has been a widening of the gap between students with relatively infrequent complaints and those with relatively frequent complaints, for instance, between students at the lowest and highest deciles of the distribution. This, in turn, suggests that reports of increasing psychosomatic complaints in adolescents cannot be explained as being primarily due to a greater inclination to report trivial complaints. Instead, the most significant increase has been in students with relatively frequent and co-occurring complaints, which in other studies have been shown to predict clinical depression [19] and suicidal ideation [20], as well as physical, emotional and behavioural impairment [11, 21, 22].

A disproportionate increase in students with relatively frequent and co-occurring complaints, and the greater polarisation of complaints that follow from this, is consistent with Norwegian [26] and British [27] studies focusing on complaints related to depression and psychological distress. This implies that health inequality understood as interindividual variations in complaints related to mental ill-health has increased. It is beyond the scope of the current study to analyse the reasons behind this increased polarisation. One possible interpretation is that the determinants of the overall increase in complaints [1, 2, 6] have affected some adolescents more than others, either due to greater vulnerability or to greater exposure to these determinants.

An important aspect of the analysis by Wickström and Lindholm is that they equate the severity of complaints with the severity of life circumstances that are presumed to cause the complaints [15]. The authors seem to assume that a complaint is trivial if it is caused by trivial circumstances in life, and vice versa, that it is severe if it is caused by severe circumstances. A key distinction is between experiencing trivial complaints, combined with ‘feeling well’ at the core, versus experiencing severe complaints and ‘not feeling well’ [15]. Combining psychosomatic complaints and their presumed psychosocial causes in one concept is at odds with common practice in survey-based research on psychosomatic complaints [2–4, 7], and the authors of the current paper make no assumptions concerning the aetiology of psychosomatic complaints.

It should be emphasised that the validity of the specific empirical results reported by researchers critical of survey-based methods [15] are not being questioned by this study. In fact, the constructivist perspective employed by these researchers, meaning that it is assumed that surveys partially construct the phenomena that they purportedly aim to measure, may be seen as incommensurable with the more ‘positivist’ perspective inherent to survey-based research, in which surveys are typically viewed as neutral tools that measure pre-existing phenomena. Moreover, as a general point, it should be stressed that qualitative investigations into how adolescents themselves understand surveys could provide important insights. However, certain key implications of the criticism of Wickström and Lindholm [15] of population-level estimates of prevalence and trends in complaints are not borne out by the data.

### Limitations

The frequency and co-occurrence of complaints as measured by the HBSC do not directly correspond to the categorisation presented by Wickström and Lindholm [15] of trivial versus severe complaints (or everyday vs. severe ‘problems’), which incorporate both actual complaints and life circumstances assumed to cause the complaints. In this sense, the observable implications investigated in this study only constitute indirect or approximate tests of the criticism presented by Wickström and Lindholm [15]. Another limitation is that the HBSC lacks specific questions about functional impairments related to the reported complaints. However, previous studies have found that these or similar complaints are indeed correlated with physical, emotional and behavioural impairment in adolescents [3, 11, 21, 22].

## Conclusions

Using three decades of HBSC data, this study has shown first that psychosomatic complaints in Swedish adolescents are distributed on a continuum with no distinct clusters of respondents and, second, that the increase in complaints has been driven by a disproportionate increase in students reporting frequent and co-occurring complaints, thereby leading to greater polarisation. The study presents a novel investigation of trends across the full distribution of responses in an instrument used to monitor psychosomatic complaints in adolescents. The results provide new evidence on the characteristics of trends in complaints and clarify how they can be understood in light of the questions that have been raised concerning the validity of survey-based measures. The results do not support recent criticism which states that estimates of psychosomatic complaints in adolescents conflate trivial with severe complaints and thereby primarily capture trivial complaints that do not reflect underlying health problems.

## Supplemental Material

sj-docx-1-sjp-10.1177_14034948221094497 – Supplemental material for Trends in adolescent psychosomatic complaints: a quantile regression analysis of Swedish HBSC data 1985–2017 Click here for additional data file. Supplemental material, sj-docx-1-sjp-10.1177_14034948221094497 for Trends in adolescent psychosomatic complaints: a quantile regression analysis of Swedish HBSC data 1985–2017 by Björn Högberg, Mattias Strandh, Klara Johansson and Solveig Petersen in Scandinavian Journal of Public Health
