# Supplementary material for: Trends in adolescent psychosomatic complaints: a quantile regression analysis of Swedish HBSC data 1985–2017
Source: Scand J Public Health. 2022 May 7;51(4):619–27. doi: 10.1177/14034948221094497 (PMC10265281; doi:10.1177/14034948221094497)
Supplement: sj-docx-1-sjp-10.1177_14034948221094497 – Supplemental material for Trends in adolescent psychosomatic complaints: a quantile regression analysis of Swedish HBSC data 1985–2017 [file sj-docx-1-sjp-10.1177_14034948221094497.docx]

**Trends in adolescent psychosomatic complaints are not driven by trivial complaints: A quantile regression analysis of Swedish HBSC data 1985–2017**

**Online supplementary materials**

**Figure S1a & S1b – Histogram of the distribution of HBSC-SCL scores. Response options ‘About every day’ & ‘More than once a week’, and ‘About once a week’ & ‘About once a month’, collapsed into two broader categories.**

HBSC-SCL: Health Behaviour in School-aged Children-Symptom Checklist. Data from the Swedish version of the HBSC survey. All years refer to surveys from 1985/1986, 1993/1994, 1997/1998, 2001/2002, 2005/2006, 2009/2010, 2013/2014 and 2017/2018.

**Figure S2 – Temporal trends across different percentiles of the HBSC-SCL. Response options ‘About every day’ & ‘More than once a week’, and ‘About once a week’ & ‘About once a month’, collapsed into two broader categories.**

HBSC-SCL: Health Behaviour in School-aged Children-Symptom Checklist. Data from the Swedish version of the HBSC survey.

**Figure S3a and S3b – Histogram of the distribution of HBSC-SCL scores, in all years combined and in 1985/86 and 2017/18 separately. Only girls.**

HBSC-SCL: Health Behaviour in School-aged Children-Symptom Checklist. Data from the Swedish version of the HBSC survey. All years refer to surveys from 1985/1986, 1993/1994, 1997/1998, 2001/2002, 2005/2006, 2009/2010, 2013/2014 and 2017/2018.

**Figure S3c and S3d – Histogram of the distribution of HBSC-SCL scores, in all years combined and in 1985/86 and 2017/18 separately. Only boys.**

HBSC-SCL: Health Behaviour in School-aged Children-Symptom Checklist. Data from the Swedish version of the HBSC survey. All years refer to surveys from 1985/1986, 1993/1994, 1997/1998, 2001/2002, 2005/2006, 2009/2010, 2013/2014 and 2017/2018.

**Figure S4a – Temporal trends across different percentiles of the HBSC-SCL. Only girls.**

HBSC-SCL: Health Behaviour in School-aged Children-Symptom Checklist. Data from the Swedish version of the HBSC survey.

**Figure S4b – Temporal trends across different percentiles of the HBSC-SCL. Only boys.**

HBSC-SCL: Health Behaviour in School-aged Children-Symptom Checklist. Data from the Swedish version of the HBSC survey.

**Figure S5a – Changes in the share of responses for each response option of each item in the HBSC-SCL between 1985/86 and 2017/18. Only girls.**

Note: The bars show ratios of the proportion of responses for each response option, with the proportion in 1985/1986 as the denominator and the proportion in 2017/2018 as the numerator.

**Figure S5b – Changes in the share of responses for each response option of each item in the HBSC-SCL between 1985/86 and 2017/18. Only boys.**

Note: The bars show ratios of the proportion of responses for each response option, with the proportion in 1985/1986 as the denominator and the proportion in 2017/2018 as the numerator.
